# Impact of Ultrasonographic Features for Thyroid Malignancy in Patients With Bethesda Categories III, IV, and V: A Retrospective Observational Study in a Tertiary Center

**DOI:** 10.7759/cureus.16708

**Published:** 2021-07-29

**Authors:** Nadir Adnan Hacim, Ahmet Akbaş, Yigit Ulgen, Talar Vartanoglu Aktokmakyan, Serhat Meric, Merve Tokocin, Onder Karabay, Yuksel Altinel

**Affiliations:** 1 General Surgery, Bağcılar Training and Research Hospital, Istanbul, TUR; 2 Surgical Oncology, Bağcılar Training and Research Hospital, Istanbul, TUR; 3 Pathology, Bağcılar Training and Research Hospital, Istanbul, TUR; 4 General Surgery, Beykent University, Istanbul, TUR

**Keywords:** thyroid gland, fine-needle aspiration, thyroid neoplasms, bethesda system, cytodiagnosis

## Abstract

Introduction: Although fine-needle aspiration biopsy (FNAB) with cytologic interpretation using the Bethesda System for Reporting Thyroid Cytopathology has been widely used for thyroid nodules, its efficiency in Bethesda categories of III, IV, and V has been questioned due to variable risk of malignancy. We aimed to evaluate the impact of radiological parameters in Bethesda category III, IV, and V for thyroid malignancy.

Methods: We performed a retrospective review of patients with Bethesda category III, IV, and V, and subsequent thyroidectomy. Demographic, ultrasonographic, and clinical variables were recorded. Independent variables for thyroid malignancy and the predictive power of imaging findings were analyzed.

Results: There were 159 patients with a mean age of 48.1±13.4 years. Hypoechogenicity of the index nodule was the most common finding in 87 patients (54.7%). There were 74 (46.5%), 34 (21.4%), and 51 patients (32.1%) with Bethesda III, IV, and V categories, respectively. There were 91 patients (57.2%) with a diagnosis of thyroid malignancy. Overall malignant pathology was detected in 18 (24.3%), 25 (73.5%), and 48 patients (94.1%) in Bethesda III, IV, and V categories, respectively (p=0.001). The presence of solitary nodule, hypoechogenicity, and solid structure of index nodule and Bethesda category IV and V were significant variables for final malignant pathology (p<0.05 for all).

Conclusion: Hypoechogenicity and solid structure in a solitary index nodule should be regarded as significant ultrasonographic findings for thyroid malignancy. Bethesda category IV and V were also significantly associated with malignancy.

## Introduction

Thyroid nodules are widespread health problems, occurring in 20-60% of the adult population's ultrasound (US) reports worldwide. Their occurrence varies depending on age, sex, and geographical location [1]. Furthermore, thyroidectomy is often performed with various indications, such as malignancy or suspicious pathologies of the thyroid gland [2]. Surgical complications of thyroid surgery, including recurrent laryngeal nerve paralysis and hypocalcemia, are serious problems. Additionally, thyroid replacement therapy will be mandatory for patients with total thyroidectomy throughout their life. In light of these facts, thyroidectomy can be avoided in patients with probably benign pathologies supported by additional clinical and imaginary findings.

Fine-needle aspiration biopsy (FNAB) with cytologic interpretation is the most common diagnostic method for evaluating thyroid nodules [3,4]. The Bethesda System for Reporting Thyroid Cytopathology (BSRTC) has been declared to help physicians refine the cytologic definitions and improve the clinical management of thyroid nodules. The Bethesda classification consists of six diagnostic categories for FNAB reporting. It is logical to accept that the Bethesda categories of I (non-diagnostic or unsatisfactory), II (benign), and VI (malignant) have more precise definitions or management strategies than the other categories. So, determination of the risk of malignancy and suggestion of optimum clinical management might be controversial, especially in the Bethesda categories of III, IV, and V [4]. Bethesda III [atypia of undetermined significance/follicular lesion of undetermined significance (AUS/FLUS)], Bethesda IV [(follicular neoplasm or suspicious for follicular neoplasm (SFN)/Hurthle cell neoplasm (SFN/HCN)], and Bethesda V (suspicious for malignancy) have variable risks of malignancy, ranging from 5% to 75% [4]. Therefore, the BSRTC can be regarded as insufficient to determine which nodules are more likely malignant.

Supportive modalities to improve the BSRTC have been studied for recent decades. Some studies on molecular markers of thyroid cancer have been conducted [5]. Despite innovations, these molecular markers still have several limitations in practical usages, such as the lack of follow-up of marker-negative nodules and their costs.

In the differential diagnosis of malignant thyroid nodules, various clinical and imaging methods can be used. Several ultrasonographic features such as microcalcifications, hypoechogenicity, irregularity, and being taller than wide can predict thyroid malignancy [6,7]. However, their use for predicting malignancy in indeterminate nodules is still questionable due to low specificity and accuracy [8,9].

In this study, we aimed to investigate the clinical and radiological parameters to improve the predictive value of Bethesda category III, IV, and V for thyroid malignancy.

## Materials and methods

Study

We performed a retrospective review of patients who underwent FNAB and subsequent thyroidectomy in Bagcilar Research and Education Hospital, Istanbul, Turkey from December 2015 through November 2019. This study was approved by the Institutional Review Board of Bagcilar Research and Education Hospital (IRB No.2020.02.1.06.024). The study was performed following the Declaration of Helsinki. Written consent could not be taken from the patients due to the retrospective design of the study.

Patients

**Figure 1:**
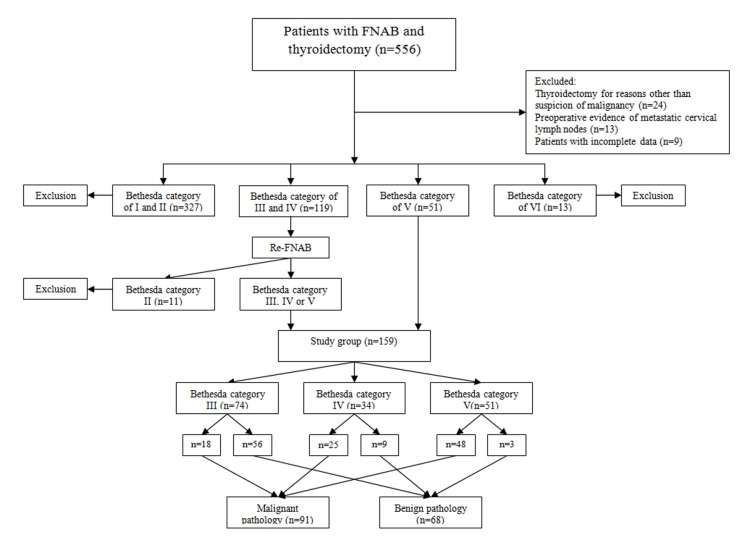
Flow chart of the study.

Among all patients with FNAB and subsequent thyroidectomy, patients with an FNAB result of Bethesda Category III (AUS/FLUS), Bethesda IV (SFN or HCN), and Bethesda V were included. As a policy, all patients with Bethesda III and IV nodules underwent repeat FNAB procedures. If the repeat FNAB results were nondiagnostic/unsatisfactory or at least Bethesda III or more, surgical treatment for histopathological confirmation was recommended. If the outcome of FNAB was benign, surgical treatment was offered in nodule size ≥4 cm, compressive symptoms, hyperthyroidism, and coexisting parathyroid pathology. For Bethesda V, surgical treatment was recommended directly. Bethesda categories of I, II, and VI, thyroidectomy for reasons other than the suspicion of malignancy, preoperative evidence of metastatic cervical lymph nodes, patients with incomplete data were excluded (Figure 1).

Variables

Information regarding demographic features (age, sex), thyroid function tests [free T3, free T4, and thyroid-stimulating hormone (TSH)], ultrasonographic findings, nodule size (mm), Bethesda category, indication for surgical treatment, operation type, as well as the final histological diagnosis was recorded using our hospital information system. The thyroid function status was classified as hypothyroid, euthyroid, or hyperthyroid based on the results of thyroid function tests. Normal ranges for free T3, free T4, and TSH were 1.71-3.71 pg/mL, 0.70-1.48 ng/dL, and 0.35-4.94 IU/mL, respectively.

Ultrasound examination

Thyroid US was performed by Esaote Color Doppler US (Model: 796FDII Yung-ho City, MAG Technology Co, Ltd., Taipei, Taiwan) by its superficial probe (model no: LA523 13-4, 5.5-12.5 MHz). The patient was in a supine position, and the neck was hyperextended. The index nodule number could be one or more depending on the radiologist's decision who performs the US. The presence of a solitary nodule and the diameter of the index nodule was recorded. All nodules were also grouped as <2 cm and ≥2 cm and analyzed accordingly. During US examination, the following features were recorded for each nodule: echogenicity (hypoechoic or hyper- and isoechoic), margin (irregular or well-bordered), structure (solid, mixed or cystic), being taller than wide in shape (absent or present), microcalcifications (absent or present), increased vascularity (absent or present) and loss of peripheral halo (absent or present) [6,10].

FNAB and cytology

Under US guidance, FNAB was performed using the General Electric Logiq Pro 200 (Model number 2270968; GE Healthcare Korea, Seongnam SI, Gyean GGI-DO, Korea) and 5.5-7.5 MHz probe. The decision of FNAB was performed based on the American Thyroid Association Guidelines for Adult Patients with Thyroid Nodules [3]. In summary, the nodules ≥1 cm with high and intermediate suspicious sonographic patterns underwent histological examination via FNAB. The nodules ≥1.5 cm with low and ≥2 cm with low suspicious sonographic patterns were also evaluated by FNAB. The slides prepared by aspiration were fixed by air drying and stained using May-Grunwald-Giemsa stains for cytological evaluation. Informed consent for FNAB was taken from all patients. All cytological and pathological examinations of thyroid pathologies were performed by one experienced cytopathologist with at least five years of experience in thyroid pathology.

The BSRTC system was used to report FNAB results as (I) nondiagnostic or unsatisfactory, (II) benign, (III) AUS or FLUS, (IV) SFN or HCN, (V) suspicious for malignancy, and (VI) malignant [4].

Statistical analysis

The final histopathological diagnosis of a surgical specimen was accepted as the reference. If the final malignant pathology was confirmed for the index nodule, the lesion was considered malignant.

Statistical analysis was performed using a statistical package (SPSS 15.0, Chicago, IL, USA). Continuous variables with and without normal distribution were presented as mean ± standard deviation and median with interquartile range (IQR) of 25-75%. Categorical variables were presented with frequencies with percentages. Student’s t-test, Mann-Whitney U test, Pearson chi-square, and Fisher’s exact test were used for univariate analysis. We analyzed the US findings in a multivariate binary logistic regression model to estimate the odds ratios (OR; 95% confidence interval). The sensitivity, specificity, false-negative and positive rates, positive and negative predictive values were calculated for each US finding. A p-value of <0.05 was considered statistically significant.

## Results

General features of the study group

There were 159 patients with a mean age of 48.1±13.4 years. The majority of the patients were female (71.7%). Demographic and clinical features are given in Table 1. During the US examination, hypoechogenicity was the most common finding in 87 patients (54.7%). Other results are summarized in Table 1.

**Table 1:** Demographic and clinical features of the study group (n=159). ^β^Mean±standard deviation; ^¥^n (%), ^µ^Median (interquartile range). US: ultrasound, FNAB: fine-needle aspiration biopsy.

Variable	Value
Age (year)^β^	48.1±13.4
Sex^¥^	
Female	114 (71.7)
Male	45 (28.3)
Radiation history^¥^	
Yes	2 (1.3)
Family history^¥^	
Yes	6 (3.8)
Status of thyroid function^¥^	
Hypothyroidism	56 (35.2)
Euthyroidism	79 (49.7)
Hyperthyroidism	24 (15.1)
Diameter of nodule (mm)^µ^	18 (11.0–30.0)
Nodule size^¥^	
<2 cm	83 (52.2)
≥2 cm	76 (47.8)
Solitary nodule^¥^	
Yes	69 (43.4)
US features^¥^	
Hypoechogenicity	87 (54.7)
Irregularity	32 (20.1)
Taller than wide	49 (30.8)
Solid structure	85 (53.5)
Microcalcifications	45 (28.3)
Loss of halo	28 (17.6)
Increased vascularity	22 (13.8)
Cervical lymph nodes	47 (29.6)
FNAB^¥^	
Bethesda III	74 (46.5)
Bethesda IV	34 (21.4)
Bethesda V	51 (32.1)
Predominant indication for surgery^¥^	
FNAB	93 (58.5)
Nodule size	7 (4.4)
Compressive symptoms	13 (8.2)
Hyperthyroidism	11 (6.9)
Coexisting parathyroid pathology	7 (4.4)
Type of surgery^¥^	
Lobectomy	12 (7.5)
Total thyroidectomy	147 (92.5)
Lymphadenectomy^¥^	
Central	8 (5.0)
Regional	12 (7.5)
Pathology^¥^	
Adenomatous nodule	16 (10.1)
Colloidal nodule	18 (11.3)
Hemorrhagic cyst	4 (2.5)
Graves’ disease	4 (2.5)
Hashimoto’s thyroiditis	14 (8.8)
Follicular adenoma	11 (6.9)
Hurthle cell adenoma	1 (0.6)
Follicular carcinoma	7 (4.4)
Papillary carcinoma	79 (49.7)
Hurthle cell carcinoma	1 (0.6)
Medullary carcinoma	4 (2.5)

Cytopathological examination revealed that there were 74 (46.5%), 34 (21.4%), and 51 patients (32.1) with Bethesda III, IV, and V categories, respectively. The result of FNAB was the predominant indication for surgical treatment in 93 patients (58.5%). Additional indications are detailed in Table 1. Total thyroidectomy was performed in 147 patients (92.5%). The final pathological analysis revealed that papillary carcinoma was present in approximately half of the patients (49.7%). Other pathological diseases are given in Table 1. Based on the final pathological diagnoses, 91 patients (57.2%) were diagnosed with thyroid malignancy.

**Figure 2:**
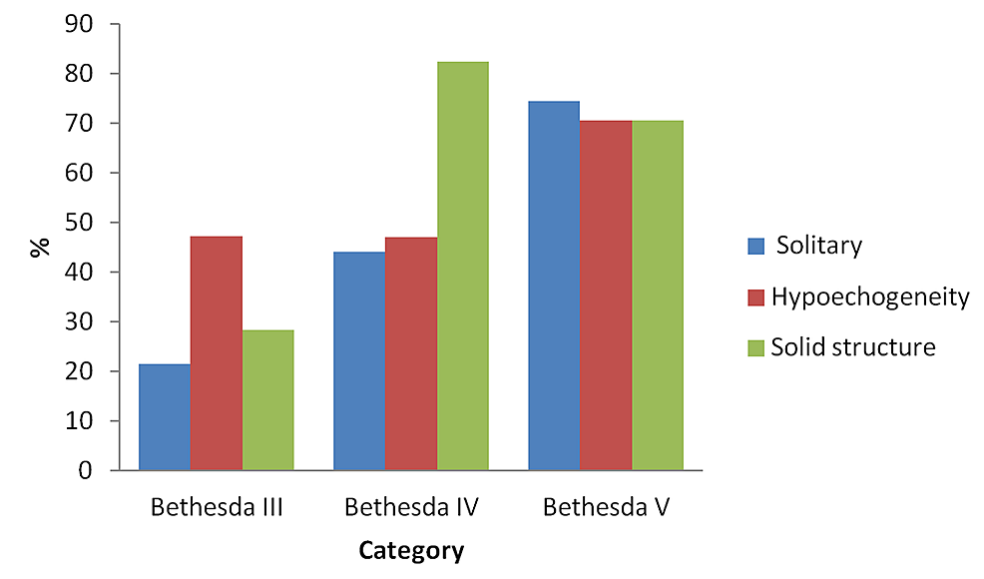
Distribution of ultrasonographic features (solitary, hypoechogenicity, solid structure) (%) in Bethesda categories of III, IV, and V.

Assessment of clinical and ultrasonographic features in Bethesda III, IV, and V categories Demographic and clinical features of the patients based on the Bethesda categories are given in Table 2. The mean age of the patients in Bethesda IV was significantly higher than that of the other two groups (p=0.017). We found the presence of a solitary thyroid nodule and its hypoechogenicity more considerably in the Bethesda V category (p<0.001 and p=0.022, respectively). The solid structure was significantly higher in patients with categories IV and V (82.4% and 70.6% of the patients, respectively, Figure 2). There were no significant differences in other demographic and clinical features between the groups (p>0.05 for all; Table 2).

**Table 2:** Association of the demographic and clinical variables with Bethesda categories. ^β^Mean±standard deviation; ^¥^n (%); ^µ^median (interquartile range). Bold font indicates statistical significance. US: ultrasound, FNAB: fine-needle aspiration biopsy.

Variable	FNAB			p-Value
	Bethesda III (n=74)	Bethesda IV (n=34)	Bethesda V (n=51)	
Age (year)^β^	45.6±13.2	53.4±13.3	48.1±128	0.017
Sex				
Female	56 (75.7)	25 (73.5)	33 (64.7)	0.394
Male	18 (24.3)	9 (26.5)	18 (35.3)	
Radiation history				
Yes	1 (1.4)	0 (0)	1 (2.0)	0.726
No	73 (98.6)	34 (100)	50 (98.0)	
Family history				
Yes	3 (4.1)	2 (5.9)	1 (2.0)	0.640
No	71 (95.9)	32 (94.1)	50 (98.2)	
Status of thyroid function				
Hypothyroidism	26 (35.1)	10 (29.4)	20 (39.2)	0.131
Euthyroidism	32 (43.2)	22 (64.7)	25 (49.0)	
Hyperthyroidism	16 (21.6)	2 (5.9)	6 (11.8)	
Diameter of nodule (mm)	20 (11.0–30.0)	22 (14.0–33.0)	17 (10.0–25.0)	0.169
Nodule size				
<2 cm	36 (48.6)	14 (41.2)	33 (64.7)	0.073
≥2 cm	38 (51.4)	20 (58.8)	18 (35.3)	
Solitary nodule				
Yes	16 (21.6)	15 (44.1)	38 (74.5)	<0.001
US features				
Hypoechogenicity	35 (47.3)	16 (47.1)	36 (70.6)	0.022
Irregularity	13 (17.6)	7 (20.6)	12 (23.5)	0.714
Taller than wide	22 (29.7)	11 (32.4)	16 (31.4)	0.958
Solid structure	21 (28.4)	28 (82.4)	36 (70.6)	<0.001
Microcalcifications	18 (24.3)	10 (29.4)	17 (33.3)	0.540
Loss of halo	11 (14.9)	7 (20.6)	10 (19.6)	0.693
Increased vascularity	10 (13.5)	6 (17.6)	6 (11.8)	0.739
Cervical lymph nodes	25 (33.8)	4 (11.8)	18 (35.3)	0.037

**Figure 3:**
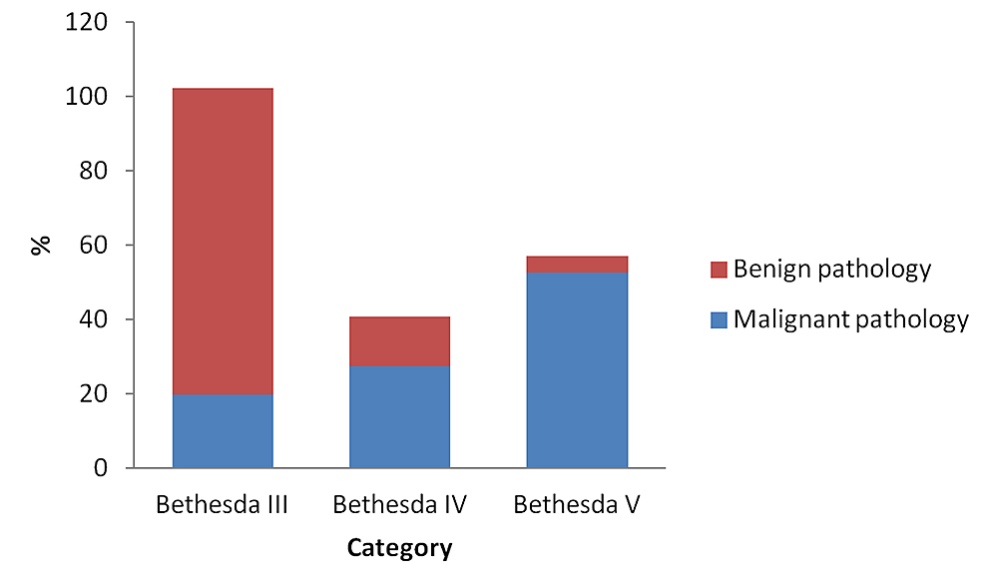
Distribution of malignant and benign thyroid pathologies (%) in Bethesda categories of III, IV, and V.

In Bethesda category III, benign pathology was detected in 56 patients (75.7%). However, malignant pathology was significantly higher in Bethesda IV (73.5%) and Bethesda V categories (94.1%), respectively (p=0.001; Figure 3 and Table 3).

**Table 3:** Distribution of pathological diagnoses in Bethesda categories. ^¥^n (%). Bold font indicates statistical significance. FNAB: fine-needle aspiration biopsy.

Pathology	FNAB^¥^			p-Value
	Bethesda III (n=74)	Bethesda IV (n=34)	Bethesda V (n=51)	
Adenomatous nodule	11 (14.9)	2 (5.9)	3 (5.9)	
Colloidal nodule	15 (20.3)	3 (8.8)	0 (0.)	
Hemorrhagic cyst	4 (5.4)	0 (0)	0 (0)	
Graves’ disease	4 (5.4)	0 (0)	0 (0)	
Hashimoto’s thyroiditis	14 (18.9)	0 (0)	0 (0)	
Follicular adenoma	7 (9.5)	4 (11.8)	0 (0)	0.001
Hurthle cell adenoma	1 (1.4)	0 (0)	0 (0)	
Follicular carcinoma	0 (0)	7 (20.6)	0 (0)	
Papillary carcinoma	16 (21.6)	17 (50)	46 (49.7)	
Hurthle cell carcinoma	0 (0)	1 (2.9)	0 (0)	
Medullary carcinoma	2 (2.7)	0 (0)	2 (3.9)	
Overall benign pathology	56 (75.7)	9 (26.5)	3 (5.9)	0.001
Overall malignant pathology	18 (24.3)	25 (73.5)	48 (94.1)	

Assessment of clinical and ultrasonographic features in malignant and benign thyroid pathologies

**Figure 4:**
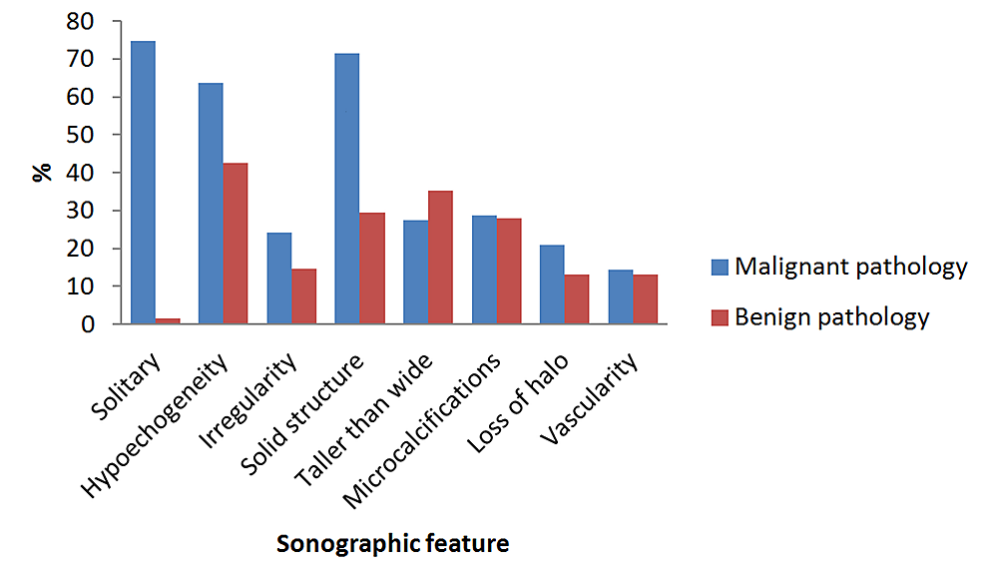
Distribution of sonographic features (%) in patients with malignant and benign pathology.

The presence of solitary nodule, hypoechogenicity, and solid structure of the index nodule and Bethesda category were the significant variables between the patients with a final malignant and benign pathology (p<0.05 for all, respectively; Table 4). Bethesda III was the most common category detected in 82.4% of the patients with benign pathology. The distribution of all sonographic features in patients with malignant and benign pathology is shown in Figure 4. There was no difference in other demographic and clinical variables between the patients with and without malignant pathology (p>0.05 for all).

**Table 4:** Comparison of demographic and clinical features of the patients with malignant and benign pathology. ^β^Mean±standard deviation, ^¥^n (%), ^µ^median (interquartile range). Bold font indicates statistical significance. US: ultrasound, FNAB: fine-needle aspiration biopsy.

Variable	Malignant pathology (n=91)	Benign pathology (n=68)	p-Value
Age (year)^β^	49.7±13.8	45.9±12.5	0.074
Sex^¥^			
Female	62 (68.1)	52 (76.5)	0.248
Male	29 (31.9)	16 (23.5)	
Radiation history^¥^			
Yes	1 (1.1)	1 (1.5)	1.0
No	90 (98.9)	67 (98.5)	
Family history^¥^			
Yes	5 (5.5)	1 (1.5)	0.240
No	86 (94.5)	67 (98.6)	
Thyroid functions^¥^			
Hypothyroidism	34 (37.4)	22 (32.4)	0.453
Euthyroidism	46 (50.5)	33 (48.5)	
Hyperthyroidism	11 (12.1)	13 (19.1)	
Diameter of nodule (mm)^µ^	18 (11.0-30.0)	20 (12.0–29.0)	0.621
Nodule size^¥^			
<2 cm	49 (53.8)	34 (50)	0.748
≥2 cm	42 (46.2)	34 (50)	
Solitary nodule^¥^			
Yes	68 (74.7)	1 (1.5)	<0.001
US features^¥^			
Hypoechogenicity	58 (63.7)	29 (42.6)	0.008
Irregularity	22 (24.2)	10 (14.7)	0.141
Solid structure	65 (71.4)	20 (29.4)	<0.001
Taller than wide	25 (27.5)	24 (35.3)	0.291
Microcalcifications	26 (28.6)	19 (27.9)	0.930
Loss of halo	19 (20.9)	9 (13.1)	0.211
Increased vascularity	13 (14.3)	9 (13.2)	0.849
Cervical lymph nodes	28 (30.8)	19 (27.9)	0.699
Bethesda III	18 (19.8)	56 (82.4)	0.001
Bethesda IV	25 (27.5)	9 (13.2)	
Bethesda V	48 (52.7)	3 (4.4)	

Considering US findings, binary logistic regression revealed only hypoechogenicity and solid structure were the independent predictors of malignancy (Table 5). 

**Table 5:** Independent risk of malignancy for US findings with binary logistic regression analysis. Bold font indicates statistical significance. US: ultrasound, OR: odds ratio, CI: confidence interval.

US finding	OR (95% CI)	p-Value
Hypoechogenicity	2.364 (1.242–4.497)	0.008
Irregularity	1.849 (0.810–4.220)	0.141
Solid structure	6.0 (3.003–11.987)	<0.001
Taller than wide	0.694 (0.353–1.368)	0.291
Microcalcifications	1.031 (0.513–2.074)	0.930
Loss of halo	1.730 (0.729–4.107)	0.211
Increased vascularity	1.093 (0.438–2.727)	0.849

Predictive statistics of US imaging findings are given in Table 6. The highest sensitivity rates were detected for solid structure and hypoechogenicity of the index nodule as 71.4 and 63.7.

**Table 6:** Precision of US features for detecting malignancy in Bethesda III, IV, and V nodules. US: ultrasound, NPV: negative predictive value, PPV: positive predictive value.

US features	Sensitivity	Specificity	False-negative rate	False-positive rate	NPV	PPV
Hypoechogenicity	63.7	57.4	36.3	42.6	54.2	66.7
Irregularity	24.2	85.3	75.8	14.7	45.7	68.8
Solid structure	71.4	70.6	28.6	29.4	64.9	76.5
Taller than wide	27.5	64.7	72.5	35.3	40.0	51.0
Microcalcifications	28.6	72.1	71.4	27.9	43.0	57.8
Loss of halo	20.9	86.8	79.1	13.2	45.0	67.9
Increased vascularity	14.3	86.8	85.7	13.2	43.1	59.1

## Discussion

This study showed that hypoechogenicity and solid structure of the solitary index nodule were significant US findings for predicting thyroid malignancy in Bethesda III, IV, and V nodules. The age of the patients in Bethesda IV was significantly higher than that of other categories. Besides, the malignant pathology rates increased as the category of the index nodule increased from Bethesda III to Bethesda V. 

In previous studies and meta-analyses, nodule size was associated with an increased risk of thyroid cancer [11-14]. However, Kamran et al. [1] reported a nonlinear association between nodule size and papillary carcinoma risk. The risk increased for nodules up to 2.0 cm. Beyond this threshold, the cancer risk remained the same. Also, follicular and other rare carcinomas were detected in larger nodules [1,15]. Kiernan and Solórzano [15] also reported the lack of association between nodule size of indeterminate lesions and malignancy risk after controlling for age and sex. Interestingly, a smaller nodule size was a significant predictor of thyroid cancer [16]. In the present study, the majority of thyroid cancer cases were papillary carcinoma. The number of other types was relatively low. In that way, we could not analyze the association between the type of thyroid cancer and the nodule size. However, grouping based on the threshold as 2 cm did not reveal any significant association. Therefore, nodule size alone should not guide to perform FNAB in these patients.

Some US features, including solid structure, microcalcification, hypoechogenicity, increased vascularity, and irregular margins, are high-risk thyroid malignancy factors [10,15,16]. According to this study, solitary nodules have also been regarded as another suspicious feature for thyroid malignancy [8]. However, the number of these features shows excellent variations in each study [17]. In the study by Norlén et al. [6], hypoechogenicity, irregular margins, and microcalcifications were significant predictors of malignancy in Bethesda III nodules. Although PPV values of all three criteria were low, the NPV for one or more of the three criteria was 98.3%. This finding meant that only 1.7% of the nodules were malignant if all three were absent. In the study by Molina-Vega et al. [16], solid component and irregular margins were independent risk factors for malignancy in Bethesda categories VI, V, and VI. In the same study, isoechoic nodules have had higher odds ratios for malignancy. Wu et al. [10] found that irregularity was the only significant criterion for all Bethesda III, IV, and V categories, contrary to our study. Li et al. [8] showed the significant association of irregular borders, solitary nodules, hypoechogenicity, being taller than wide, and microcalcification to malignancy in Bethesda III and IV categories. In their study, the overall malignancy rate was 54.2%. In the present study, solitary nodule, hypoechogenicity, and solid structure were significant predictive findings for thyroid malignancy. We also found that the distribution of these ultrasonographic findings shows variations according to different Bethesda categories, i.e., hypoechogenicity as the most common finding in Bethesda category III contrary to a solid nodule with hypoechogenicity in Bethesda category IV. Besides, we also thought that the PPV and NPV values for each US criterion were poor to reach a reliable diagnosis for the FNAB results of all Bethesda III, IV, and V nodules. There was heterogeneity of the thyroid pathologies, unstandardized US findings, and different Bethesda categories in each study. Therefore, it is difficult to conclude that any US criterion can exclude or confirm malignancy effectively.

According to the previously published studies, there are different malignancy rates for Bethesda III, IV, and V categories. In the original report of the BSRTC, the risk of malignancy has been reported as 5-15%, 15-30%, and 60-75% for Bethesda III, IV, and V, respectively [4]. In literature, the overall malignancy rates varied from 9.3% to 48.9% for Bethesda III [2,6,9,17-20]. Several authors proposed subcategorization or subgroupings based on the cytological findings of FNAB [18,20]. They thought that using such systems helps physicians overcome confusion problems that originated from the BSRTC system. In the present study, these rates were 24.3%, 73.5%, and 94.1%, respectively. The higher rates of thyroid malignancy, especially for Bethesda category IV, may be related to the feature of our institution as the referral tertiary center for thyroid diseases in its district area. As an explanation for the difference between malignancy rates, selection bias for thyroid cancer by including the patients treated surgically alone may be accused.

The results of FNAB in the thyroid gland aspirates are also affected by the technique, the expertise, and experience of cytological reading. A standard criterion has been advocated to avoid such errors due to inadequate specimen quality, interpretation of unsatisfactory specimens, and lack of diagnostic category standardization [21]. All these scientific backgrounds cause the development of standardized reporting systems like the BSRTC system. An acceptable degree of agreement between several examiners with varied cytopathology experience has been shown using this system [22]. The authors also mentioned that the BSRTC system is usable even by a beginner in cytopathology. The specimens with adequate cellularity and benign categories caused a higher concordance degree between different pathologists [23]. So, there might be some degree of difficulties for other indeterminate varieties. Such a situation may be regarded as a bias that should be considered for the FNAB results.

For the last decades, molecular testing has been used for guiding the treatment of indeterminate thyroid pathologies [5,15]. However, we cannot have the ability to perform molecular testing for thyroid pathologies due to financial problems. 

The relatively small sample size and retrospective design were significant limitations. The number of patients in this study might be inadequate to reach more meaningful and significant results. Inclusion of the cases only with thyroidectomy might cause selection bias to reach more convincing conclusions.

## Conclusions

Hypoechogenicity and solid structure in a solitary index nodule should be a critical US finding for thyroid malignancy. Hypoechogenicity was the most common finding in Bethesda category III. A solid nodule with hypoechogenicity was frequently seen in Bethesda category IV. No significant association was detected between the diameter of the nodule and thyroid cancer. Bethesda category IV and V were also significantly associated with malignancy. However, nodule size alone may not be regarded as a predictive feature for malignancy. We suggest that predictive preoperative sonographic characteristics such as solitary nodule, hypoechogenicity, and solid structure may be considered for surgical treatment of patients with indeterminate thyroid nodules.
